# Hybrid virtual reality object lifting matches real-world object lifting

**DOI:** 10.64898/2026.04.13.718283

**Published:** 2026-05-04

**Authors:** Catherine Anne Sager, Jackson Zenti, Michelle Marneweck

**Affiliations:** 1 Department of Human Physiology, University of Oregon, Eugene, Oregon, United States; 2 Institute of Neuroscience, University of Oregon, Eugene, Oregon, United States; 3 Phil and Penny Knight Campus for Accelerating Scientific Impact, Eugene, Oregon, United States

**Keywords:** Hybrid virtual reality, object manipulation

## Abstract

Clinical deafferentation underscores the fundamental role of proprioception in motor control, but chronic sensory loss also drives long-term compensatory strategies that complicate mechanistic inference. Because proprioceptive reliability is difficult to manipulate experimentally, its contribution to skilled control remains unclear. Virtual reality (VR) with controlled visuo-proprioceptive offsets provides a promising model of proprioceptive unreliability that induces sensory reweighting toward vision during conflict. This VR-offset framework has advanced our understanding of vision-dominant control under proprioceptive unreliability in reaching tasks. It remains unknown how the motor system responds to proprioceptive unreliability during skilled object manipulation. Unlike reaching, manipulation requires anticipatory force/torque control that accounts for trial-to-trial variability in digit position; these policies are learned within a few trials, yet changes in object dynamics produce anterograde interference that increases with greater repetition before the dynamics switch. Although vision, tactile cues, and prior experience support these features, the role of proprioceptive reliability remains unresolved. Hybrid-VR, which pairs real object interaction with virtual visual feedback, offers a way to address this gap. Before introducing offsets, we must establish that hybrid-VR without offsets reproduces the hallmark behaviors highlighted above. Here, we compared real-world object manipulation with hybrid-VR object manipulation where participants (*N* = 15) lifted and stabilized an object with an asymmetric mass distribution. Across real-world and hybrid-VR conditions, the rate of anticipatory force control, trial-to-trial position-force adjustment, and switch-related interference were indistinguishable. These results demonstrate that hybrid-VR reproduces hallmark features of dexterous manipulation, providing a foundation for future studies isolating proprioceptive reliability.

## NEW AND NOTEWORTHY

Hybrid virtual reality (VR), combining a real object interaction with immersive VR, preserves core features of real-world object manipulation, including rapid anticipatory force learning, trial-by-trial coordination of digit position and force, and repetition-induced interference. This hybrid-VR approach maintains natural sensorimotor control while allowing for controlled manipulation of visual information. This validated framework provides a new tool to isolate how proprioceptive reliability shapes skilled object manipulation.

## 1. INTRODUCTION

Proprioception plays a fundamental role in motor control, providing information about limb position, movement, and force that is essential for skilled action (1-3). Evidence from clinical deafferentation demonstrates that proprioceptive loss disrupts motor performance (4); however, chronic sensory loss also induces long-term compensatory strategies that complicate mechanistic inference about proprioception’s causal role in motor control (5). As a result, despite decades of study, the contribution of proprioceptive reliability to skilled motor behavior remains incompletely understood.

One promising approach to isolating proprioceptive contributions to motor control is the use of virtual reality (VR) to experimentally manipulate the relationship between vision and proprioception. By introducing controlled visuo-proprioceptive offsets, VR can render proprioceptive signals unreliable without eliminating them, thereby inducing sensory reweighting toward vision during conflict. This framework has substantially advanced our understanding of vision-dominant control policies during reaching to virtual targets (5-9). However, it remains unknown how the motor system responds to proprioceptive unreliability during skilled object manipulation, a domain that places distinct and more stringent demands on sensorimotor control.

Unlike reaching, object manipulation requires precise control of fingertip position and forces (10-12) together with rapid online adjustments in response to trial-to-trial variability in digit placement (particularly in the case of object manipulations requiring torque forces) (13,14). This control is learned quickly, often within only a few trials, as individuals learn to scale forces to object properties such as mass and mass distribution (15,16). These policies can eventually generalize when object dynamics change (e.g., a switch in the massdistribution direction), but adaptation is not immediate. Instead, early post-switch behavior often reflects anterograde interference (17,18) where force and torque outputs remain biased toward the pre-switch condition. Importantly, this carryover is magnified when the pre-switch condition is extensively repeated (*i.e.*, repetition-induced anterograde interference) (15,19). Although vision (13,18,20-25), haptics (26-29), and prior experience (10,11,30-32) are known to support anticipatory and online force control, the contribution of proprioception remains largely unexplored.

VR offers a potential route to address this gap by pairing real-world object interactions with programmable offsets between visual and proprioceptive information. However, before such offsets can be meaningfully introduced, it is necessary to establish if a hybrid-VR object manipulation task without an offset can faithfully reproduce the core behavioral signatures of real world dexterous control. Without this validation, any observed effects of visuo-proprioceptive conflict could reflect VR-specific artifacts unrelated to the manipulation of proprioceptive reliability.

Here, we tested whether a hybrid-VR object lifting task recapitulates core features of real-world object manipulation: rapid learning of anticipatory force control for specific object properties, trial-to-trial variability in digit position and forces, and susceptibility to repetition-induced anterograde interference. Participants lifted a visually symmetric object with an asymmetric mass distribution, either viewed directly or through VR, while preventing tilt toward the weighted side. By comparing performance across real-world and hybrid-VR conditions, we asked whether a hybrid-VR object lifting task preserves the control mechanisms underlying dexterous manipulation. Establishing this provides a necessary foundation for future studies using VR-based visuo-proprioceptive offsets to isolate the contribution of proprioceptive reliability to object manipulation.

## 2. MATERIALS AND METHODS

**Figure 1:**
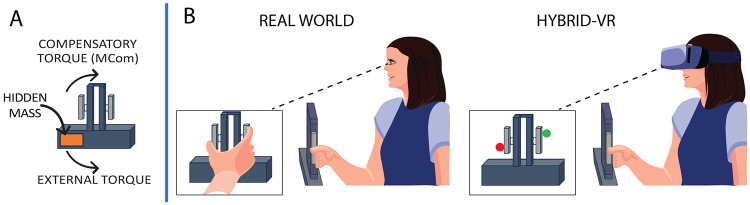
Object and Task Design. A) Inverted T-shaped object with a concealed off-center mass. Participants had the task goal of minimizing tilt by generating a compensatory torque ($M_{\text{Com}}$) that opposes the object’s external torque. B) Lifting the inverted T-shaped object with the right index finger and thumb in the real world (left) and hybrid virtual reality (VR) (right). In the real world, participants saw the object and their grasping hand/digits throughout the reach, grasp, and object lift (left box); in hybrid-VR, participants saw a visual representation of the object in VR and their grasping digits thumb: red marble; index finger: green marble) throughout the reach, grasp, and lift (right box).

Participants completed a well-established asymmetric-load object lifting task (13-15,18,19,25,31-35) in real-world and hybrid-VR conditions: in the hybrid-VR condition, they physically lifted a real object while viewing it in VR. In this task, participants reached, grasped, and lifted an inverted T-shaped object with a concealed off-centered mass while preventing it from tilting toward the weighted side. Successful performance required anticipatory force control, achieved by generating a compensatory torque (Figure 1A) at lift onset through coordinated digit placement, grip force, and lift force. We tested whether learning of anticipatory force control, trial-to-trial adjustments in digit position and lift forces, and repetition-induced anterograde interference are expressed similarly in real-world and hybrid-VR conditions (Figure 1B), thus establishing hybrid-VR as a valid platform for future investigations of proprioceptive reliability during object manipulation.

### 2.1 Participants

Fifteen healthy right-handed young adults (median age: 23, range: 18-35, standard deviation = 2.8; 10 females) participated in this study. The study and all its procedures were approved by the University of Oregon Institutional Review Board, and all participants gave written informed consent.

### 2.2 Apparatus

The T-object was fabricated with aluminum rods and plates. The object’s vertical column (height: 10.2 cm; width: 1.0 cm; depth: 3.4 cm) had smooth elongated grasp surfaces (height: 7.0 cm; width: 0.3 cm; depth: 4.1 cm; between grasp distance: 4.6 cm) with 400 grain sandpaper attached on either side of the grasp surfaces. A lead cylinder (height: 4.5 cm; diameter: 2.4 cm; mass: 215 g) was concealed in the horizontal base (height: 3.8 cm; width: 32.3 cm; depth: 3.8 cm). The total mass of the object was 650 g with an external torque of 250 Nmm. A three-camera motion tracking system (Precision Point Tracking System; Worldviz) with a frame rate of 150 Hz (camera resolution: 1,280 × 1,024 VGA) was used to track the vertical height of the object. The system’s spatial accuracy within a 3 × 3 × 3 m volume was ≤ 1 mm. Two near-infrared LED markers were affixed securely to the covers on the horizontal base of the object to monitor the vertical position of the object. Grip forces and torque that were applied to the grip surfaces of the object were recorded at a frequency of 500 Hz through force/torque transducers (Mini27 Titanium, ATI Industrial Automation, NC). These force transducers were attached between each grip surface and the vertical column of the T-shaped object. Grip force, load forces, and torque, with resolutions of 0.03 N, 0.015 N, and 0.375 Nmm were measured by the transducers. Data were filtered using a fourth-order low-pass Butterworth filter with a cutoff frequency of 5 Hz.

For the VR task, participants wore an HTC VIVE headset (2160 × 1200 resolution, PenTile OLED display; HTC VIVE, Xindian District, New Taipei City, Taiwan) equipped with integrated headphones (HTC VIVE Tracker (3.0) Developer Guideline ver. 1.1, 2021) and sampled at a rate of 120 Hz. A 3D virtual environment was rendered using the Unity3D game engine (v2018.4.10f1; Unity Technologies, San Francisco, USA) with the SteamVR plugin. The HTC VIVE tracking system used SteamVR infrared tracking to record the real-time position and orientation of two near-infrared LED markers affixed to the inverted T-shaped object, as well as two additional markers placed on the participant’s right index finger and thumb. In the VR display (Figure 1B), the finger markers were represented as marble-sized spheres—green for the index finger and red for the thumb (Figure 1B). The custom VR environment included a white floor, a virtual desk with a tape box indicating where to replace the object, a keyboard button, and the inverted T-shaped object used for the lifting task. Auditory cues were presented through the headset’s headphones.

### 2.3 Experimental Procedures

In both real-world and hybrid-VR conditions, participants were seated in a chair at a comfortable height such that their right forearm and upper arm made a right angle while resting on the table. They were instructed to keep their left arm from resting in their lap. At the start of each trial, participants pressed a keyboard button (home button) with their right index finger, 20 cm from the T-shaped object that they would interact with throughout the task. A “left” or “right” audio cue informed them which side of the inverted T-shaped object would be heavier. A beep occurred 1 s after the left/right side audio cue that instructed participants to reach to lift the object without tilting it. Participants were informed that they could grasp the object anywhere along the elongated grasp surfaces. The object was lifted to a height of 11 cm. A second beep (2.50 s after the first) instructed participants to set the object back down in a taped square on the table and return their right index finger to its start position on the keyboard button. If the object rolled more than 5 degrees to either side, an error tone would sound. Participants were told to complete both tasks at a natural pace and to attempt to lift the object without tilting it to the weighted side at all times.

To examine whether real-world and hybrid-VR object lifting show similar effects of anticipatory force control learning and repetition-induced anterograde interference, we manipulated the number of trial repeats in the object-use task before implementing a switch in the object’s center of mass (CoM). Participants were exposed to five sets of 1 pre-switch trial and 1 post-switch trial and five sets of 5 pre-switch trials and 1 post-switch trial, giving a total of 40 trials (30 pre-switch lifts, 10 post-switch lifts). Both real-world and hybrid-VR conditions were completed by the same participants (within-subjects design) in two separate sessions (48 hours apart). The order of environments (real-world *vs.* hybrid-VR), the starting location of the CoM (left vs. right), and pre-switch trial condition (5 vs. 1) were counterbalanced between subjects.

### 2.4 Data Processing

1. Grip force mean (${\text{GF}}_{\text{mean}}$) at lift onset is the instantaneous average in grip force of each digit in Newtons (N). This was calculated using a numerical averaging method:
$${\text{GF}}_{\text{mean}}=\left({\text{GF}}_{\text{thumb}}+{\text{GF}}_{\text{index}}\right)∕2$$
2. Lift force difference (${\text{LF}}_{\text{diff}}$) at lift onset is the variation in the tangential component of the force produced by each digit (N).
$$\text{Lift force difference}\;({\text{LF}}_{\text{diff}})={\text{LF}}_{\text{thumb}}-{\text{LF}}_{\text{index}}$$
  where higher thumb than index finger lift force shows positive values and a higher index finger than thumb lift force shows negative values. Larger differences indicate a more asymmetric lift force sharing pattern, whereas a zero value indicates a symmetric lift force sharing pattern.
3. Center of pressure (COP) at lift onset is the measure of digit position defined as the point of contact of each digit on the grip surface relative to the center of the transducer (in mm). This was computed using the formula:
$${\text{COP}}_{\text{digit}}=\left(Tx_{\text{digit}}-({\text{LF}}_{\text{digit}}\times \text{grip surface thickness})\right)∕{\text{GF}}_{\text{digit}}$$
  where $Tx_{\text{digit}}$ is the digit torque in the frontal plane (Nmm). The difference between thumb and index finger placement was used to identify grip configuration:
$$\text{Center of pressure difference}\;({\text{COP}}_{\text{diff}})={\text{COP}}_{\text{thumb}}-{\text{COP}}_{\text{index}}$$
  where positive values indicate higher thumb than index finger COP and negative values indicate higher pointer finger than thumb COP. Larger differences indicate a more asymmetric, non-collinear grip configuration, whereas a zero value indicates a symmetric, collinear grip configuration.
4. Compensatory moment or torque ($M_{\text{Com}}$) at lift onset is the anticipatory torque generated by the digits measured in Newton millimeters (Nmm) to counter the external torque of the object. This was computed using the formula:
$$M_{\text{Com}}=({\text{LF}}_{\text{diff}}\times (d∕2))+({\text{GF}}_{\text{mean}}\times {\text{COP}}_{\text{diff}})$$
  where d is the width between both grip surfaces (4.6 cm). A positive $M_{\text{Com}}$ indicated a clockwise moment, and a negative $M_{\text{Com}}$ indicated a counterclockwise moment.

For participants who began the task with the CoM on the right, $M_{\text{Com}}$, ${\text{LF}}_{\text{diff}}$, and ${\text{COP}}_{\text{diff}}$ values were multiplied by −1 to avoid the statistical complication caused by different signs of $M_{\text{Com}}$ when manipulating an object with a left *vs.* a right CoM (15,19,20).

### 2.5 Data Analyses

To quantify within-CoM learning of anticipatory force control, we focused on the 5 pre-switch lifts in each condition (real world, hybrid-VR). Because performance did not differ across blocks (*p* > 0.05), we collapsed across blocks and, for each participant and condition, fit a least-squares linear regression of $M_{\text{Com}}$ on trial number (1-5). The resulting slope coefficient was used as the individual learning-rate metric (i.e., change in $M_{\text{Com}}$ per trial). Slopes were then compared between real world and hybrid-VR using a paired-samples *t*-test.

To assess trial-by-trial coordination between digit positioning and digit lift force, we examined the relationship between ${\text{COP}}_{\text{diff}}$ and ${\text{LF}}_{\text{diff}}$ across the five pre-switch lifts for each participant in each condition (real-world and hybrid-VR). For each participant, Pearson’s correlation coefficients (r) were calculated between ${\text{COP}}_{\text{diff}}$ and ${\text{LF}}_{\text{diff}}$, and then Fisher *r*-to-*z* transformed for statistical testing. Participants’ individual Fisher *z* values were compared between real-world and hybrid-VR conditions using a paired-samples *t*-test to understand whether the strength of digit positioning and digit lift force differed across conditions. Group-level mean r values were reported to describe the overall magnitude of these correlations.

To assess whether repetition-induced anterograde interference is equivalently expressed for real-world and hybrid-VR conditions, we used a two-way repeated measures ANOVA to examine the effects of condition (real-world vs. hybrid-VR) and pre-switch repetition (1 vs. 5) on post-switch $M_{\text{Com}}$. Because performance did not differ across blocks (p > 0.05), we collapsed across blocks.

## 3. RESULTS

### 3.1 No Difference in Anticipatory Torque Learning Between Real World and Hybrid-VR Manipulation

**Figure 2:**
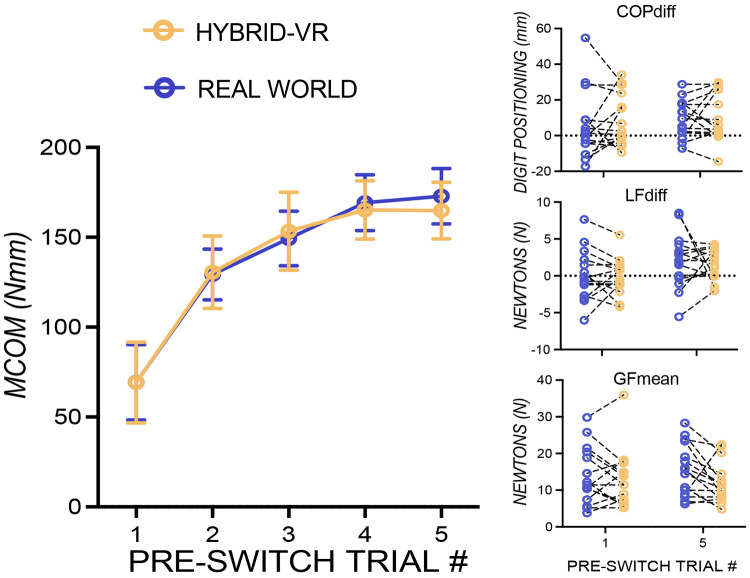
Learning Rate of Anticipatory Torque Control in Real-World and Hybrid Virtual Reality (VR) Object Manipulation. Group mean compensatory moment ($M_{\text{Com}}$) aggregated across five blocks of pre-switch trials evaluate how rapidly performance ($M_{\text{Com}}$) improves per trial in both real-world vs. hybrid-VR conditions. Increasing experience with a given center of mass led to improved anticipatory torque control in both real-world (solid purple line) and hybrid-VR (solid yellow line) conditions. Individual data points for the first and fifth pre-switch lifts show the components of $M_{\text{Com}}$—center of pressure difference (${\text{COP}}_{\text{diff}}$), lift force difference (${\text{LF}}_{\text{diff}}$), and mean grip force (${\text{GF}}_{\text{mean}}$)—have comparable patterns across environments, with no meaningful differences between real-world and hybrid-VR conditions. Error bars are +/− 1 std. error.

Figure 2 shows learning-related changes in anticipatory torque control across pre-switch trials in both real-world and hybrid-VR conditions. $M_{\text{Com}}$ increased significantly with trial number repeats in both real world (*β* = 22.61, *p* = 0.0005) and hybrid-VR (*β* = 24.69, *p* < 0.0001), indicating improved learning in both conditions. Critically, slopes did not differ between conditions (*t* (14) = −0.39, *p* = 0.71), indicating that the hybrid-VR object lifting task preserves the learning dynamics of anticipatory force control observed during real world manipulation. Additionally, the components of $M_{\text{Com}}$ (${\text{COP}}_{\text{diff}}$, ${\text{LF}}_{\text{diff}}$, and ${\text{GF}}_{\text{mean}}$) showed comparable patterns across environments, suggesting similar coordination of digit forces and positions in both real-world and hybrid-VR conditions (all *p*’s > 0.05; Figure 2).

### 3.2 Similar Trial-By-Trial Variation in Digit Positions and Lift Force Partitioning in Real World and Hybrid-VR Manipulation

**Figure 3:**
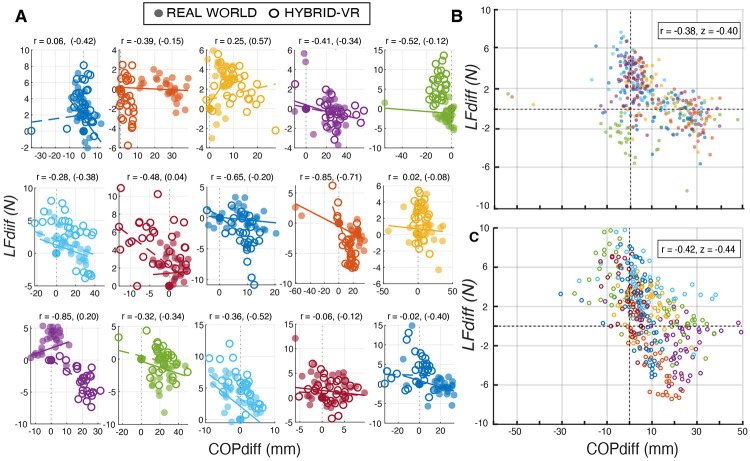
Trial-by-Trial Digit Position and Lift Force Partitioning Scatterplots for Real World and Hybrid Virtual Reality (VR) Manipulation. A) Individual participants’ trial-by-trial scatterplots and Pearson correlation coefficients (*r*) (hybrid-VR in parentheses) showing moderate to strong negative relationships between lift force difference (${\text{LF}}_{\text{diff}}$) and center-of-pressure difference (${\text{COP}}_{\text{diff}}$) during object lifting in both real-world (closed circles) and hybrid-VR (open circles) conditions. Different axis scales were used across participants for visualization purposes. Each subplot depicts data from a single participant, with colors identifying individuals. B) Group-level real world scatterplot showing the negative relationship between ${\text{LF}}_{\text{diff}}$ and ${\text{COP}}_{\text{diff}}$ across participants. C) Group-level hybrid-VR scatterplot showing the negative relationship between ${\text{LF}}_{\text{diff}}$ and ${\text{COP}}_{\text{diff}}$ across participants. In both B and C, points are colored by participant.

To assess whether the hybrid-VR object lifting task has hallmark variation in trial-to-trial control processes observed during real world manipulation, we quantified and compared the relationship between ${\text{LF}}_{\text{diff}}$ and ${\text{COP}}_{\text{diff}}$ across pre-switch lifts for each of these conditions. Figure 3A shows individual participants’ trial-by-trial correlations between ${\text{LF}}_{\text{diff}}$ and ${\text{COP}}_{\text{diff}}$ for both real-world and hybrid-VR conditions. Most participants showed moderate, negative correlations in both real world (mean *r* = −0.38, 95% CI [−0.75, 0.17]; Figure 3B) and hybrid-VR manipulation (*r* = −0.42, 95% CI [−0.77, 0.12; Figure 3C]), with no significant differences between these conditions (*t (*14) = 0.33, *p* = 0.74). These findings indicate that the hybrid-VR object lifting task preserves the coordinated trial-by-trial control policies that characterize dexterous object manipulation in real world settings.

### 3.3 Presence of Repetition-Induced Anterograde Interference in the Lift Component of the Real World and Hybrid-VR Manipulation Tasks

**Figure 4:**
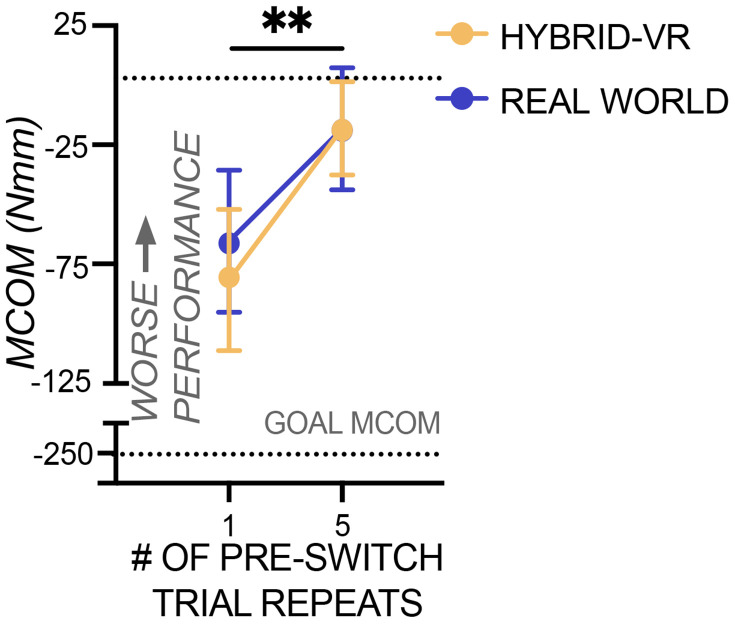
Repetition-Induced Anterograde Interference Present in Both Real World and Hybrid Virtual Reality (VR) Manipulation. Post-switch compensatory moment (MCom) following five pre-switch trials. Values in the real-world condition is shown in purple lines with filled circles and that in the hybrid-VR in yellow lines with filled circles. The goal MCom is indicated at −250 Nmm. Increasing the number of pre-switch trials before the switch led to larger post-switch errors in both real-world and hybrid-VR conditions, demonstrating repetition-induced anterograde interference. Asterisks indicate statistical significance (** *p* < 0.01). Error bars are +/− 1 std. error.

Figure 4 shows that repetition-induced anterograde interference was magnified in post-switch trials as the number of pre-switch repetitions increased. The effect of pre-switch repetition on post-switch $M_{\text{Com}}$ was significant (*F* (1, 28) = 10.43, *p* = 0.0032, *η*_p_^2^ = 0.27) for both real-world and hybrid-VR conditions, with no interaction effect (*p* > 0.05). Importantly, this effect was present in both real-world and hybrid-VR conditions, with no significant differences between conditions (*p* > 0.05). These results replicate prior findings on repetition-induced anterograde interference and extend them by demonstrating that the effect generalizes to a hybrid-VR object lifting task, validating hybrid-VR as a platform for studying dexterous object manipulation.

## 4. DISCUSSION

In this study, we tested whether a hybrid-VR environment elicits core features of dexterous object manipulation observed in the real world. Specifically, we examined whether hybrid-VR preserves rapid learning of anticipatory force control, coordinated trial-to-trial variability in digit position and force, and susceptibility to repetition-induced anterograde interference. Participants performed an asymmetric object lifting task both in a real-world setting and hybrid-VR environment in which they manipulated a physical object while viewing it virtually. Consistent with our hypotheses, hybrid-VR reproduced the core behavioral signatures of real-world skilled object manipulation. Participants showed similar rates of anticipatory force learning, comparable trial-to-trial coupling between digit position and force, and equivalent repetition-induced anterograde interference across environments.

Much like prior real-world studies of asymmetric object lifting (10,16,26,30), we observed rapid learning of anticipatory force control in the real-world condition. This learning rate was preserved in hybrid-VR, where participants adjusted their compensatory torque and digit forces across the first few lifts following a change in mass distribution, demonstrating predictive updating consistent with established models of sensorimotor memory. In addition, previous work has shown that variability in digit placement is not random but systematically covaries with force output from trial to trial (13,14). We replicated this coordinated digit position–force coupling in the real-world condition and found that this relationship was maintained in hybrid-VR. Participants flexibly modulated digit forces to compensate for variability in contact position, indicating preservation of the underlying control policy governing multi-digit coordination. Finally, repetition-induced anterograde interference, in which increased prior experience with one mass distribution slows adaptation to a new distribution (15,17,19,36), was present in both environments, with comparable repetition-induced error magnitudes across conditions. Together, these findings demonstrate that hybrid-VR manipulation preserves the core behavioral features of skilled object manipulation described in the real-world literature. Performance in hybrid-VR therefore reflects the same control mechanisms engaged during physical object interaction, rather than artifacts introduced by the virtual context.

Establishing the validity of hybrid-VR for object manipulation is an important step because it enables experimental manipulations that are difficult or impossible to achieve in physical environments. In the hybrid-VR set up, participants interact with a real object, meaning that proprioceptive signals related to limb position and movement and tactile signals generated by fingertip contact arise from natural object mechanics, while visual information about the object is delivered through the virtual display. This configuration allows researchers to systematically manipulate visual feedback of the object’s position or orientation while leaving the underlying somatosensory signals generated by the interaction unchanged. As a result, hybrid-VR provides a controlled framework for introducing visuo-proprioceptive offsets during object manipulation, which can be used to effectively reduce the reliability of proprioceptive information while preserving natural interaction with the object. Studies applying visuo-proprioceptive offsets in reaching tasks have consistently shown that when visual and proprioceptive information mismatch, behavior tends to be visually guided (37-40). This bias towards vision has been interpreted as reliability-based sensory reweighting, in which the nervous system downweights proprioceptive signals when their reliability is reduced relative to vision (41). However, these approaches have rarely been applied to dexterous object manipulation. Because the present findings demonstrated that hybrid-VR preserves the core behavioral signatures of anticipatory force control and sensorimotor memory, future studies can use this framework to introduce controlled visuo-proprioceptive offsets and directly test how proprioceptive reliability contributes to object manipulation.

Prior work has begun to examine object interaction in virtual environments, but important differences in task demands and experimental design limit direct comparison with real-world dexterous manipulation. Cai et al. (2022) showed that reach-to-grasp kinematics more closely resemble real-world performance when participants interact with a physical object while viewing both the hand and object in a virtual display, highlighting the importance of visual context and physical interaction. However, this work focused on movement kinematics and did not assess whether control mechanisms underlying dexterous manipulation, such as anticipatory force scaling or trial-by-trial coordination between digit position and force, are preserved in virtual environments.

More direct comparisons for anticipatory control come from Liu et al. (2022) and Günter et al. (2026), who examined object lifting using virtual objects with device-mediated force feedback. These studies report slower trial-by-trial learning and differences in force scaling relative to prior real-world findings (Fu et al. 2010). However, several methodological differences, including the absence of direct within-subject real-world comparisons, make it difficult to isolate the effects of the virtual environment itself. In Liu et al. (2022) and Günter et al. (2026), the object lifting task was rendered in 3D but displayed on a 2D monitor rather than through an immersive headset, which may alter the interpretation of visual feedback during object interaction. In addition, both Liu et al. (2022) and Günter et al. (2026) required lifting objects with smaller torque magnitudes than those in the real-world comparison dataset, likely reducing the magnitude of error signals, and a wider range of center-of-mass conditions, which may increase task complexity and anterograde interference (44,45). Together, these factors make it difficult to attribute slower adaptation and differences in force scaling to virtual environments per se rather than to differences in visual presentation and task structure.

In contrast, our hybrid-VR paradigm combined immersive 3D visual feedback with interaction with a physical object and included a direct within-subject comparison to real-world performance. Under these conditions, we did not detect differences in the learning rate of force control and scaling of digit forces and positions. Critically, beyond learning rate, we show that hybrid-VR preserves hallmark features of dexterous manipulation, including trial-by-trial coupling between digit position and force and susceptibility to repetition-induced anterograde interference. Together, these findings suggest that when key features of the task closely match real-world conditions, virtual environments can preserve the hallmark control policies supporting skilled object manipulation.

The present study was not designed to evaluate longer-term generalization across extended practice. Generalization effects in asymmetric object lifting often emerge after more extensive training (24,32,33,46), and future work using longer protocols will be needed to determine whether hybrid-VR also preserves these longer-timescale learning and transfer processes.

Although the present study focused on validating hybrid-VR as a research tool, the ability to manipulate visual information while preserving natural somatosensory signals may also have broader applications for studying sensorimotor integration in populations with sensory or motor impairments. In particular, controlled visuo-proprioceptive offsets could be used to examine how changes in proprioceptive reliability influence anticipatory force control and sensorimotor memory during object manipulation, an aspect of motor behavior that has been difficult to isolate using purely physical tasks. Additionally, visual information can be systematically perturbed to reduce its reliability, allowing researchers to investigate how the motor system adapts when vision is less dependable. Such approaches may help clarify how different sensory modalities contribute to skilled manipulation when one source of information becomes unreliable, although this possibility remains to be tested in future work.

In conclusion, hybrid-VR reproduces core features of dexterous object manipulation, including rapid learning of anticipatory force control, coordinated trial-to-trial variability in digit position and force, and susceptibility to repetition-induced anterograde interference. By demonstrating that these behavioral signatures are preserved when visual information is delivered through VR while somatosensory feedback arises from interacting with a real object, the present work establishes hybrid-VR as a validated framework for studying visuo-proprioceptive interaction during dexterous object manipulation. This validation provides the critical foundation needed future studies that introduce controlled visuo-proprioceptive offsets to directly test how proprioceptive reliability shapes anticipatory force control and sensorimotor memory during skilled manual actions.

## Data Availability

All data will be made available upon reasonable request. Codes can be found on our *Motor Skill Lab* GitHub for public availability.

## GLOSSARY

CoM: Center of Mass
COPdiff: Center Of Pressure Difference
GFdiff: Grip Force Difference
LFdiff: Lift Force Difference
MCom: Compensatory torque
N: Newtons
Nmm: Newton millimeters
VR: Virtual Reality
